# Molecular and Functional Characterization of Peptidoglycan Recognition Proteins OfPGRP-A and OfPGRP-B in *Ostrinia furnacalis* (Lepidoptera: Crambidae)

**DOI:** 10.3390/insects13050417

**Published:** 2022-04-28

**Authors:** Zengxia Wang, Wan Zhou, Baohong Huang, Mengyuan Gao, Qianqian Li, Yidong Tao, Zhenying Wang

**Affiliations:** 1 College of Agriculture, Anhui Science and Technology University, Fengyang 233100, China; bhh826@163.com (B.H.); gaomytryhard@163.com (M.G.); qian_11030112@163.com (Q.L.); yidongtao2022@163.com (Y.T.); 2 State Key Laboratory for Biology of Plant Diseases and Insect Pests, MOA—CABI Joint Laboratory for Bio-Safety, Institute of Plant Protection, Chinese Academy of Agricultural Science, Beijing 100193, China; 3 College of Resource and Environment, Anhui Science and Technology University, Fengyang 233100, China; eliauk1221@163.com

**Keywords:** innate immunity, bacterial infection, *Ostrinia furnacalis*, PGRPs

## Abstract

**Simple Summary:**

The Asian corn borer, *Ostrinia furnacalis* (Guenée), is the most destructive lepidopteran insect pest of corn (*Zea mays* L.) in China. Pathogenic microorganisms play an important role in the population control of the Asian corn borer. Although microorganisms can cause the death of *O. furnacalis*, an immune response also occurs as an attempt to fight off and eliminate invading pathogens. If the molecular mechanism of interaction between *O. furnacalis* and pathogenic bacteria is clarified, the lethal effect of pathogenic microorganisms can be better exerted by inhibiting the natural immune response of *O. furnacalis*. As an important member of the pattern-recognition receptor family, peptidoglycan recognition protein (PGRP) plays a key role in the insect innate immune response. In this study, we cloned two PGRP genes from *O. furnacalis* and analyzed their spatiotemporal expression. In combination with bacterial induction experiments, we revealed the immune signal recognition pathway involved in the two proteins. The results of this study deepen the understanding of the natural immune response of *O. furnacalis* and provide new ideas for better utilization of pathogenic microorganisms in biological control of the Asian corn borer.

**Abstract:**

Peptidoglycan recognition proteins (PGRPs) are important components of insect immune systems, in which they play key roles. We cloned and sequenced two full-length PGRP, named *OfPGRP-A* and *OfPGRP-B*, from the Asian corn borer, *Ostrinia furnacalis*. These two genes comprise open reading frames of 658 and 759 bp, encoding proteins of 192 and 218 amino acids, respectively. qPCR showed that *OfPGRP-A* and *OfPGRP-B* are prominently expressed in the midgut of *O. furnacalis* fourth instar larvae. After inoculation with *Staphylococcus aureus* and *Bacillus thuringiensis*, the expression of *OfPGRP-A* was significantly upregulated, whereas the expression of *OfPGRP-B* was enhanced after inoculation with Escherichia coli. This suggests that *OfPGRP-A* mainly recognizes Gram-positive bacteria and may participate in the Toll signaling pathways, while *OfPGRP-B* identifies Gram-negative bacteria and may participate in Imd signaling pathways. Our results provide insights into the roles of PGRPs in *O. furnacalis* immune function and a foundation for using pathogens for the biological control of *O. furnacalis*.

## 1. Introduction

Innate immunity is the first defense in insects and mammals against bacterial, fungal, viral, and parasitic infections. Identifying invading pathogens is key to triggering the host immune responses. Peptidoglycan recognition protein (PGRP), a member of the pattern-recognition receptor family, can recognize pathogen-associated molecular patterns present on the pathogen surface. After recognition, the Toll signaling pathway, Imd pathway, JAK–STAT pathway, reactive oxygen metabolism, and a melanization reaction are selectively activated to eliminate pathogens [1,2,3].

PGRP was first found in the blood of *Bombyx mori*, and then in mammals (humans and mice) [4]. More than 100 PGRP family members have been identified [5]. These proteins are highly conserved, containing at least one conserved PGRP domain, which is similar to *N*-acetylmuramic acid-L-alanine amidase [6]. PGRPs have been detected in some insects, mollusks, and vertebrates but not in plants, nematodes, and aphids [7]. PGRPs can be divided into two categories according to their molecular size: short PGRPs contain a signal peptide but no transmembrane domain, whereas long PGRPs have a signal peptide and predicted transmembrane domain [8,9]. Insect PGRPs are generally expressed in immune organs. Some of these PGRPs are constitutively expressed, whereas other PGRPs are inducible. For example, in *Drosophila melanogaster*, although PGRP-S is secreted in different parts of the body, it is mostly expressed in immune organs, particularly after microbial infection. *PGRP-SB1*, *SC2*, and *SD* are highly expressed in the fat body, *PGRP-SA* is expressed in the epithelial tissue, and *PGRP-SC* is specifically expressed in viscera [10].

PGRPs play important roles in identifying invading pathogens and regulating the innate immune responses, and they can directly kill invading pathogens. PGRPs can recognize pathogenic microorganisms and activate the Toll signaling pathway and Imd pathway, which induces the synthesis of antimicrobial peptides. These two pathways are activated by Lys-type and DAP-type peptidoglycans (PGNs), respectively [11]. Dm PGRP-SA and Dm PGRP-SD in *D. melanogaster* bind with Gram-negative bacteria binding protein 1 to activate the Toll signaling pathway [12]. Dm PGRP-LC and Dm PGRP-LE of *D. melanogaster* can recognize DAP-PGN on the cell wall of Gram-negative bacteria, and the PGRP functional area outside the cell membrane binds to PGN to activate the Imd signaling pathway [13]. Different PGRPs share partly conserved structures but exhibit varied functions and expression patterns. Numerous studies have demonstrated how PGRPs in the model insects *D. melanogaster* and *B. mori* recognize bacteria, initiate immune responses, and participate in signaling pathways, whereas relatively few studies have focused on other lepidopterans.

The Asian corn borer, *Ostrinia furnacalis* (Guenée), is the most destructive lepidopteran insect pest of corn (*Zea mays* L.) in China [14]. It is widely distributed in the Asia Pacific region, where it has been reported to cause 20% to 80% yield losses in the Philippines, and 10% to 30% yield losses in China [15]. The larvae of *O. furnacalis* can bore into stalks and ear shanks, feeding on whorl leaves and young kernels, thus resulting in yield losses. The use of entomopathogens for controlling *O. furnacalis* has recently received increasing attention [16,17]. However, research on entomopathogens, including their recognition by the host immune system and role in triggering the innate immune response, remains limited.

Initially, two PGRPs were identified by analyzing full-length transcriptome sequences of pathogen-induced *O. furnacalis*. After sequence alignment, these genes were named *OfPGRP-A* (Gene ID:ON152884) and *OfPGRP-B* (Gene ID: ON152885). In this study, we conducted cloning and bioinformatics analysis of these two genes. Based on the tissue expression patterns of *OfPGRP-A* and *OfPGRP-B* after bacterial inoculation, we explored the role of the two genes in the innate immune response. We predicted the functions of *OfPGRP-A* and *OfPGRP-B* proteins and their involvement in immune signal pathways. This study provides a theoretical basis for using microbial pesticides to control Asian corn borer and for developing more efficient and environmentally friendly pesticides.

## 2. Materials and Methods

### 2.1. Insect Rearing and Bacteria Culture

*O. furnacalis* larvae were reared at 26 ± 1°C, RH = 80% ± 5%, and a photoperiod of 16 L: 8 D. The larvae were fed on a semi-artificial diet as described by Zhou [18]. The larval instar was identified according to head width.

The bacteria used in this experiment were the Gram-positive bacteria *Staphylococcusaureus* and *Bacillus thuringiensis* and the Gram-negative bacterium *Escherichia coli*. These three bacterial strains were purchased from the Institute of Microbiology, Chinese Academy of Sciences (Beijing, China). The bacteria were activated and then cultured at 37 °C and 200 rpm until the OD_600_ reached around 1. After centrifugation at 5000× *g* for 5 min at 4 °C, the supernatant was removed. The bacteria were collected, washed three times with sterile phosphate-buffered saline (pH 6.4), and resuspended to the required bacterial concentration. After autoclaving at 121 °C for 20 min, they were kept at −20 °C until use.

### 2.2. Total RNA Extraction and cDNA Synthesis

The TRIzol method was used to extract total RNA from *O. furnacalis*. First, 1% agarose gel electrophoresis was used to detect the quality of the total RNA, then a Nanodrop 2000 (Thermo Scientific, Waltham, MA, USA) was used to detect the concentration and purity of the total RNA, and an Agilent 2100 bioanalyzer (Agilent Technologies, Santa Clara, CA, USA) was used to determine the RNA integrity to ensure the quality of the RNA samples. Finally, high-quality RNA samples were reverse transcribed to synthesize cDNA using a TransScript One-step gDNA Removal and cDNA Synthesis SuperMix kit (TransGen Biotech, Beijing, China).

### 2.3. cDNA Cloning of OfPGRP-A and OfPGRP-B

According to the transcriptome sequencing results of *O. furnacalis*, two PGRP gene sequences were obtained. Based on sequence alignment and phylogenetic tree analysis, these genes were named *OfPGRP-A* and *OfPGRP-B*. Based on the cDNA sequences of *OfPGRP-A* and *OfPGRP-B*, primers were designed using Primer Premier 5.0 software (Premier Biosoft, Rockville, MD, USA) and DNAMAN5.0 (LynnonBioSoft, San Ramon, CA, USA), respectively (Table 1). The cDNA that was obtained from the fourth instar larvae at 24 h after injecting bacteria (a mixture of *S. aureus*, *B. thuringiensis*, and *E. coli.*) into *O. furnacalis* was used as a template to amplify the full-length sequence of two PGRPs. The 50-μL reaction system contained 1 µL cDNA template, 1 µL each of upstream and downstream primers (10 mmol/L), 25 µL Taq PCR Master Mixblue dye (2x), and 22 µL ddH_2_O. The PCR conditions were as follows: 94 °C for 3 min, 94 °C for 30 s, 60 °C for 30 s, and 72 °C for 1 min for 30 cycles, followed by 72 °C for 10 min. The PCR product was detected by 1% agarose gel electrophoresis. The target band was recovered using an AxyGen DNA gel recovery kit (Union City, CA, USA) and ligated into the pGEM-T Easy vector (Promega, Beijing, China). The ligation product was transformed into E. coli DH5α competent cells, and positive clones were screened using PCR with M13 universal primers. Finally, the samples were sent for sequencing to Sangon Biotech Co., Ltd. (Nanjing, China).

### 2.4. Sequence Analysis of OfPGRP-A and OfPGRP-B

We spliced and evaluated the sequenced *O. furnacalis* PGRP genes to obtain complete cDNA sequences. ExPASy (https://web.expasy.org/compute_pi/, accessed on 10 April 2022) was used to predict the molecular weight and isoelectric point of the proteins. SMART (http://smart.embl-heidelberg.de/, accessed on 10 April 2022) was used to analyze functional domains in the protein sequences. The online software SignalP 5.0 (https://services.healthtech.dtu.dk/service.php?SignalP-5.0, accessed on 10 April 2022) was used to analyze signal peptides. TMHMM Server 2.0 (https://services.healthtech.dtu.dk/service.php?TMHMM-2.0, accessed on 10 April 2022) was used to analyze the transmembrane domain. We downloaded the PGRP sequences of other insects from NCBI, used Clustal X2.0 for homology alignment analysis, and used Jalview software 2.10 for multiple sequence alignment editing. We also used MEGA 7.0 software for neighbor-joining analysis to construct a phylogenetic tree, with bootstrapping 1000 times for testing.

### 2.5. Spatiotemporal Expression Profiles of OfPGRP-A and OfPGRP-B

We collected *O. furnacalis* larvae from the 1st–5th instars and dissected 4th instars to collect samples from different tissues (hemolymph, fat body, midgut, and epidermis). Sixty 4th instar *O. furnacalis* larvae were randomly selected, their stomachs and feet were disinfected with 70% alcohol, and they were placed on ice for 5 min for freezing and anesthesia. Next, we sterilized the larvae’s gastropod using a sterilized insect needle, gently squeezed the gastropod to drop the hemolymph on parafilm, and this sample was placed in a 1.5 mL centrifuge tube. The epidermis was dissected, and the midgut, fat body, and body wall were separately collected. The hemolymph was centrifuged at 4 °C and 12,000× *g* for 30 min, and the supernatant was collected. All tissue samples were stored at −80 °C until use.

Primer Premier 5.0 software was used to design quantitative primers (Table 1). A Takara TB Green Premix Ex Taq kit (Shiga, Japan) and ABI ViiA7 real-time fluorescent quantitative PCR machine (Applied Biosystems, Foster City, CA, USA) were used to detect the expression levels of *OfPGRP-A* and *OfPGRP-B* in different stages and different tissues of the larvae. qPCR reaction consists of a system (20 μL): 1 μL cDNA template, 1 μL each upstream and downstream primers (10 mmol/L), 1 μL ROX Reference Dye II, 10 μL TB Green Premix Ex Taq, and 6 μL ddH_2_O. The program was set according to the RR420A manual as follows: 95 °C for 30 s, 95 °C for 5 s, and 60 °C for 34 s for 40 cycles, followed by 95 °C for 15 s, 60 °C for 1 min, and 95 °C for 15 s. *Ostrinia furnacalis RPL-18* was used as an internal reference gene [19].

### 2.6. OfPGRP-A and OfPGRP-B Expression after Bacterial Induction

Fourth instar larvae of *O. furnacalis* of the same size were selected and divided into a control group, phosphate-buffered saline injection group, *S. aureus* injection group, *E. coli* injection group, and *B. thuringiensis* injection group. Thirty larvae were injected for each treatment as described by Sun and Bai [20]. A microinjector was used to draw 5 µL of inactivated bacterial solution (around 3.0 × 10^6^ cells/mL); this solution was injected into the larval gastropod. After injection, the larvae surfaces were disinfected with 70% alcohol. The larvae were then reared at 26 ± 1 °C, RH = 80 ± 5%, and a photoperiod of 16 L: 8 D.

Five larvae were collected for dissection at 2, 4, 8, 12, 24, and 48 h after injecting the bacteria, and different tissue samples were collected for quantitative analysis. Three replicates were used for each treatment. The collection method and qPCR program were the same as those described in Section 2.5, and the sample RNA extraction and cDNA synthesis steps were performed as described in Section 2.2.

### 2.7. Data Analysis

The relative expression levels of *OfPGRP-A* and *OfPGRP-B* in *O. furnacalis* at different instars, in different tissues, and after induction with different bacteria were calculated using the 2^−∆∆Ct^ method [21]. Differences in expression between samples or treatments were analyzed using analysis of variance (ANOVA). SPSS16.0 software (SPSS, Inc., Chicago, IL, USA) was used for statistical analysis. Illustrator software Origin 8.0 (OriginLab, Northampton, MA, USA) was used to prepare illustrations.

## 3. Results

### 3.1. Molecular Characteristics and Phylogenetic Analysis of OfPGRP-A and OfPGRP-B cDNA Sequence

**Figure 1:**
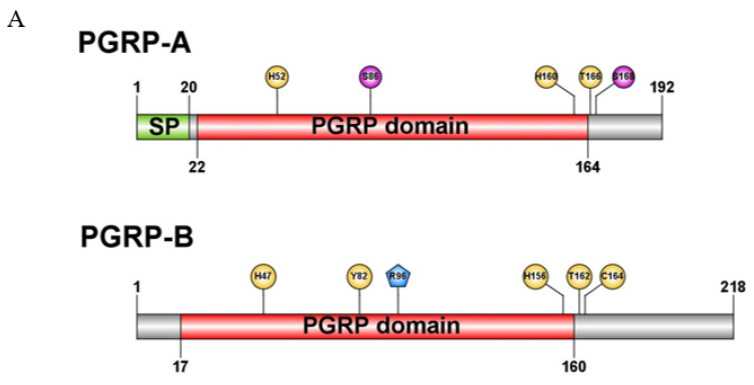

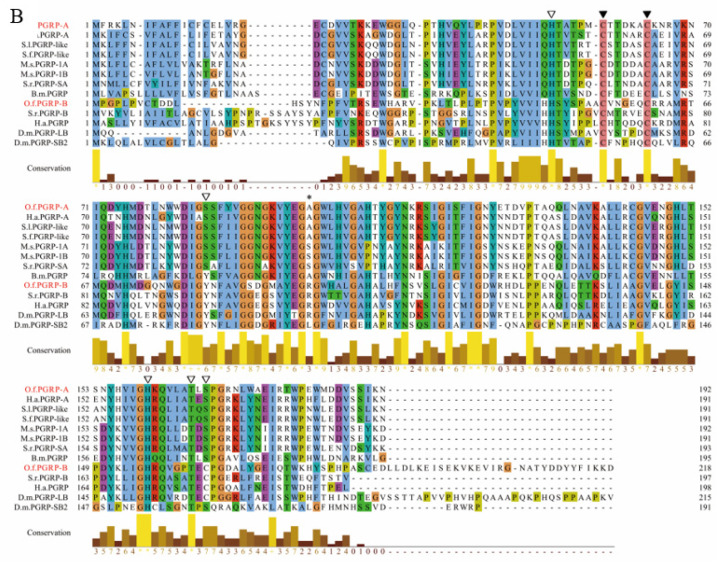
(**A**) Schematic presentation of the protein structure of *OfPGRP-A* and *OfPGRP-B*. Signal peptides (SP) and peptidoglycan recognition protein (PGRP) domain are indicated with green and red, respectively. Yellow circles and purple circles represent key amino acid sites that determine zinc ion/amidase activity and mutation sites, respectively. Blue pentagon represents key DAP-type amino acid sites. (**B**) Multiple-sequence alignment of *OfPGRP-A* and *OfPGRP-B* with other insect PGRPs. Ha: *Helicoverpaarmigera*; Ms: *Manduca sexta*; Sl: *Spodopteralitura*; Sf: *Spodopterafrugiperda*; Sr: *Samiaricini*; Bm: *Bombyx mori*; Dm: *Drosophila melanogaster*. ▽ Key amino acid sites that determine zinc ion/amidase activity, ▼ conserved cysteine sites, and * key DAP-type amino acid sites.

The full-length sequences of *O. furnacalis OfPGRP-A* and *OfPGRP-B* were obtained by PCR amplification. The complete cDNA of *OfPGRP-A* consisted of 658 bp and encoded a protein of 192 amino acids (Figure 1A). The predicted molecular mass of the *OfPGRP-A* protein was 21.79 kD, with an estimated pI of 8.20, and lacked a transmembrane domain but had a PGRP domain (amino acids 22–164), Ami2 domain (amino acids 33–170), and a *N*-terminal signal peptide comprising 20 amino acids. The open reading frame of *OfPGRP-B* was 759 bp and encoded a protein of 218 amino acids (Figure 1B). The predicted molecular weight of the encoded protein was 24.44kD with a pI of 5.68, and it had no transmembrane domain nor signal peptide but had a PGRP domain (amino acids 17-160) and an Ami2 domain (amino acids 29-166). These structural characteristics indicated that *OfPGRP-A* and *OfPGRP-B* are short-form PGRPs.

The PGRPs of *Helicoverpaarmigera*, *Manduca sexta*, *Spodopteralitura*, *Spodopterafrugiperda*, and *Samiaricini* were selected for multiple-sequence alignment with OfPGRP-A and OfPGRP-B amino acid sequences (Figure 1A,B). The conserved regions of insect PGRPs are very consistent, and the domains are highly conserved. OfPGRP-A and OfPGRP-B contain two and three conserved cysteine residues, respectively. Some insects contain the H–Y–H–T–C structure, which is necessary for forming the amidase active sites with amidase activity. The sequence alignment results showed that both *O. furnacalis* OfPGRP-A and OfPGRP-B contained the five key amino acid residues required for PGRP/amidase activity. However, in the sequence of OfPGRP-SA, tyrosine is mutated to serine and cysteine is mutated to serine, suggesting that the protein encoded by OfPGRP-A lacks amidase activity. In contrast, OfPGRP-B retains the five key amidase active sites H–Y–H–T–C, indicating that the encoded protein has amidase activity. Additionally, OfPGRP-B contains arginine residues and a DAP-type PGN recognition site, indicating that OfPGRP-B can recognize DAP-type PGN.

**Figure 2:**
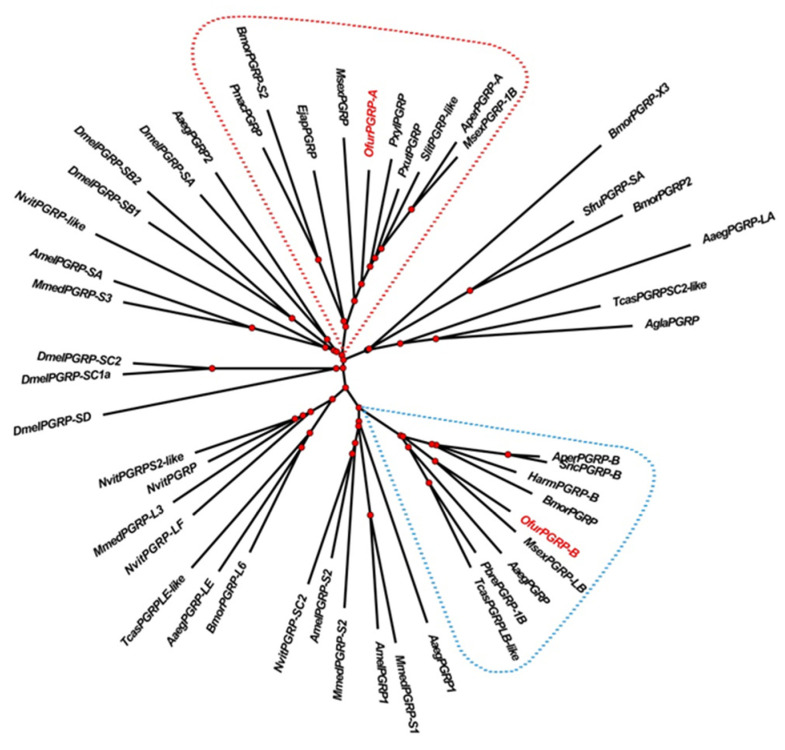
Phylogenetic tree of OfPGRP and PGRPs from other species. Bmor: *Bombyx mori*; Sfru: *Spodopterafrugiperda*; Aaeg: *Aedes aegypti*; Tcas: *Triboliumcastaneum*; Agla: *Anoplophoraglabripennis*; Aper: *Antheraeapernyi*; Sric: *Samiaricini*; Harm: *Helicoverpaarmigera*; Msex: *Manduca sexta*; Pbre: *Protaetiabrevitarsis*; Mmed: *Microplitis mediator*; Amel: *Apis mellifera*; Nvit: *Nasoniavitripennis*; Dmel: *Drosophila melanogaster*; Pmac: *Papiliomachaon*; Ejap: *Eumeta japonica*; Pxyl: *Plutellaxylostella*; Pxut: *Papilio Xuthus*; Slit: *Spodopteralitura*.

The phylogenetic tree of *O. furnacalis* PGRP and that of other species constructed using MEGA 7.0 software and online website modification is shown in Figure 2. Cluster analysis showed that OfPGRP-A and the *P. xylostella* PGRP are closely related. OfPGRP-B clustered with PGRP-Bs from other insects and is closely related to that in *M. sexta*, with an amino acid sequence conservation of 96%.

### 3.2. Spatiotemporal Expression of OfPGRP-A and OfPGRP-B

**Figure 3:**
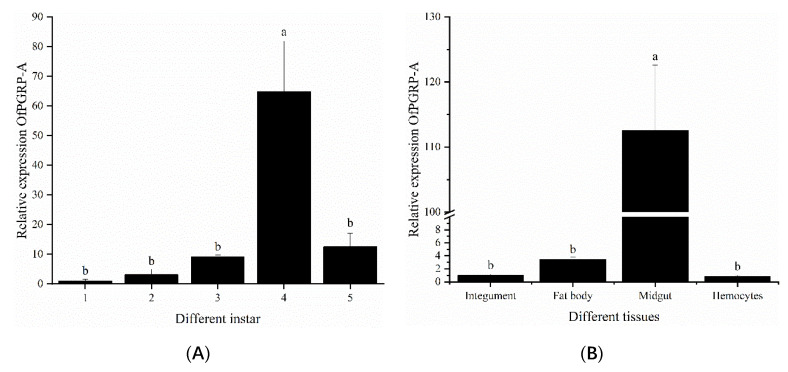
Relative expression levels of *OfPGRP-A* in different developmental stages (**A**) and adult tissues (**B**) of *Ostrinia furnacalis*. The small letters represent a signifificant difference (one-way ANOVA, followed by Tukey’s test as post hoc, *p* < 0.05).

**Figure 4:**
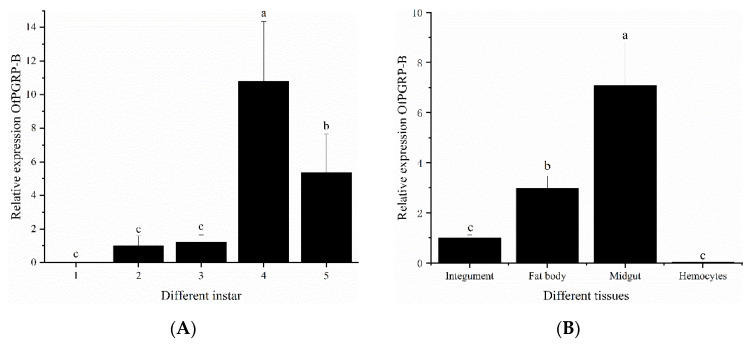
Relative expression levels of *OfPGRP-B* in different developmental stages (**A**) and adult tissues (**B**) of *Ostrinia furnacalis*. The small letters represent a signifificant difference (one-way ANOVA, followed by Tukey’s test as post hoc, *p* < 0.05).

We used *PRL-18* as an internal reference gene to analyze the expression patterns of PGRP in different stages and different tissues of *O. furnacalis* larvae. The expression levels of *OfPGRP-A* and *OfPGRP-B* were highest in 4th instar larvae, showing significantly higher levels compared with those in other instars (*p* < 0.05). The expression level of *OfPGRP-A* in 4th instar larvae was 55-fold higher than that in 1st instar larvae, and *OfPGRP-B* showed the lowest expression level in 1st instar larvae, with the expression being almost undetectable (Figure 3A and Figure 4A). *OfPGRP-A* was expressed in all larval tissues. Expression was highest in the midgut, which showed significantly higher levels compared with all other tissues (*p* < 0.05), followed by expression in the fat body. Expression was lowest in the hemolymph; expression in the midgut was nearly 140-fold higher than that in the hemolymph. *OfPGRP-B* was also highly expressed in the midgut at significantly higher levels than in the epidermis and hemolymph, indicating that the expression of *OfPGRP* is tissue-specific (Figure 3B and Figure 4B).

### 3.3. Expression of OfPGRP-A and OfPGRP-B after Bacterial Inoculation

**Figure 5:**
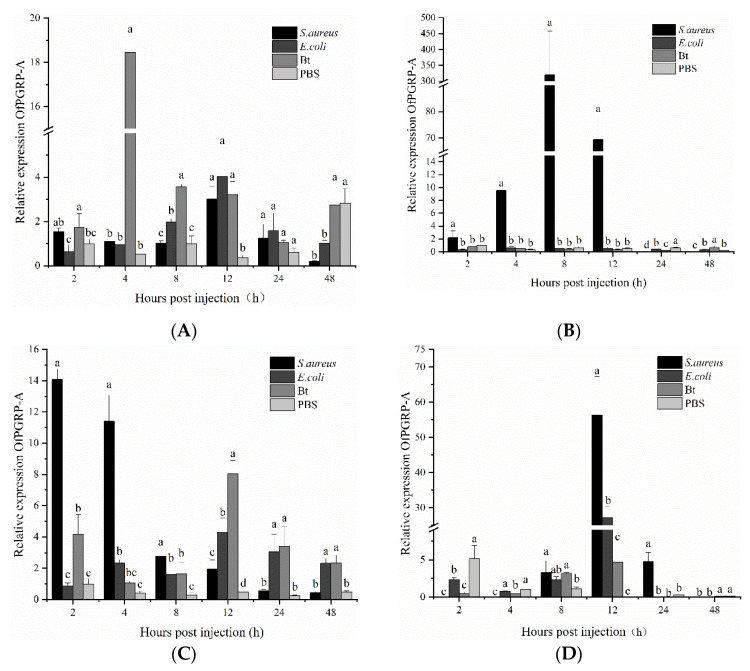
Expression levels of *OfPGRP-A* in different tissues of 4th instar larvae of *Ostrinia furnacalis* after injection with different pathogens: (**A**) epidermis; (**B**) hemolymph; (**C**) fat body; (**D**) midgut. PBS: PBS buffer; Bt: *Bacillus thuringiensis*; Staph: *Staphylococcus aureus*; RIL: *Escherichia coli*. The small letters represent a signifificant difference (one-way ANOVA, followed by Tukey’s test as post hoc, *p* < 0.05).

Inoculation of the three bacteria caused changes in the expression level of *OfPGRP-A* in *O. furnacalis* larvae (Figure 5). In the epidermis, the expression of *OfPGRP-A* was highest at 4 h after *B. thuringiensis* injection (35-fold higher than that in the control (*p* < 0.05)), after which the expression gradually decreased. *OfPGRP-A* was also significantly upregulated at multiple time points after *S. aureus* induction. In the hemolymph, *OfPGRP-A* was significantly activated by *S. aureus*. The expression levels at 2, 4, 8, and 12 h after induction were significantly higher than those in the control and after other bacterial treatments. At 8 h after treatment, the expression of *OfPGRP-A* reached the highest level (*p* < 0.05; 205-fold higher than that in the control). In the fat body, *OfPGRP-A* expression was upregulated at all time points. At 2 and 4 h after *S. aureus* injection, the expression level was significantly higher than in the other groups (*p* < 0.05), after which the expression level decreased. The expression level of *OfPGRP-A* following *B. thuringiensis* induction reached the highest level after 12 h. After induction by *E. coli*, the expression level of *OfPGRP-A* was upregulated, although the increase was not as significant as that observed with the other two bacteria. In the midgut, the expression of *OfPGRP-A* reached the highest level at 12 h after each of the three bacterium injections, with the expression being significantly higher than that in the control (*p* < 0.05). The most significant increase in *OfPGRP-A* expression was induced by *S. aureus*. Some PGRPs act as pattern recognition receptors that recognize and bind to foreign pathogens and then activate the Toll and Imd pathways. Lysine-type PGN mainly activates the Toll pathway, while the DAP-type PGN activates the Imd pathway [22,23]. According to our results, *OfPGRP-A* expression was significantly increased after induction by the Gram-positive bacteria *S. aureus* and *B. thuringiensis*. This gene may be involved in activating the Toll pathway.

**Figure 6:**
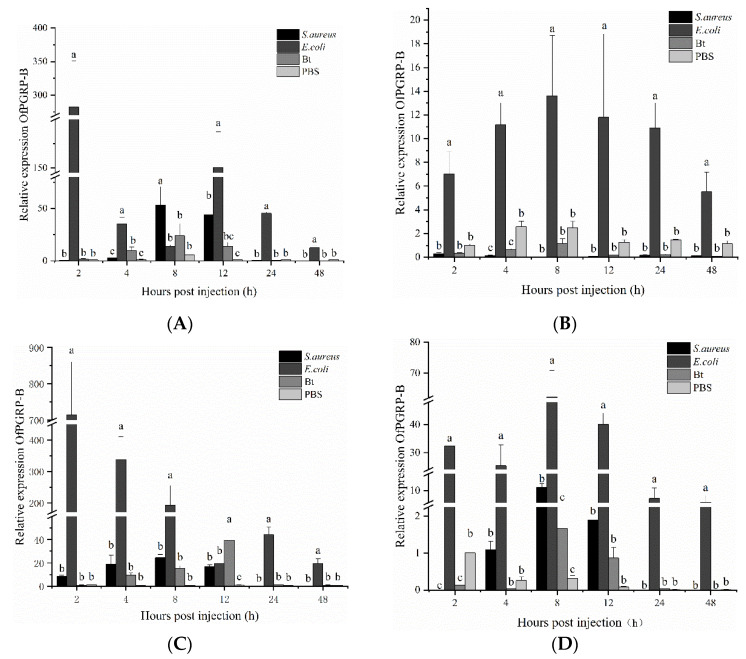
Expression levels of *OfPGRP-B* in different tissues of 4th instar larvae of *Ostrinia furnacalis* after injection with different pathogens: (**A**) epidermis; (**B**) hemolymph; (**C**) fat body; (**D**) midgut. PBS: PBS buffer; Bt: *Bacillus thuringiensis*; Staph: *Staphylococcus aureus*; RIL: *Escherichia coli*. The small letters represent a signifificant difference (one-way ANOVA, followed by Tukey’s test as post hoc, *p* < 0.05).

The expression level of the *OfPGRP-B* gene in *O. furnacalis* larvae was higher than that in the control group and other bacterial treatment groups at various time points and in different tissues after *E. coli* injection (Figure 6). In the epidermis, the expression of *OfPGRP-B* reached the highest level at 2 h after *E. coli* induction, showing significantly higher expression than that in the control group (*p* < 0.05), after which it decreased. In the hemolymph, after *E. coli* induction, the expression of *OfPGRP-B* increased, reaching the highest expression level at 8 h, and then decreased. Compared with *S. aureus* and *B. thuringiensis*, there is a significant difference in expression (*p* < 0.05). *OfPGRP-B* expression was significantly upregulated in the fat body after *E. coli* induction compared with that in the control group at all time points (*p* < 0.05). The fold-increase was particularly significant in the first eight hours. *S. aureus* and *B. thuringiensis* could also increase *OfPGRP-B* expression, but to lower levels than that induced by *E. coli* injection. In the midgut, after injection of the three bacteria, the induction effect of *E. coli* was the most significant, followed by *S. aureus*. Compared with the control, the expression of *OfPGRP-B* significantly differed at 4, 8, and 12 h after induction, reaching the highest level at 8 h. According to these results, the expression of *OfPGRP-B* increased significantly after induction by the Gram-negative bacteria *E. coli*. This gene may activate the Imd pathway.

## 4. Discussion

Insects possess innate immunity against invading microbial pathogens. PGRPs in insects play important roles in recognizing bacterial infections. These proteins can specifically recognize PGNs on bacterial surfaces, which activate the Toll and Imd pathways as well as downstream immune responses [7,12]. In recent years, the functions of various members of the PGRP family have been determined in insects; however, these studies mainly focused on model insects, such as *D. melanogaster* and *B. mori*. Two PGRP gene sequences (*OfPGRP-A* and *OfPGRP-B*) were screened from the sequence data of the transcriptome of *O. furnacalis* larvae by sequence comparison and evolutionary analysis. In this study, the full-length cDNA sequences of *OfPGRP-A* and *OfPGRP-B* were successfully cloned, the sequence characteristics of the genes were analyzed using bioinformatics software, and their spatiotemporal and pathogen-induced expression patterns were detected using qPCR.

Insect PGRP can be divided into short PGRP-S and long PGRP-L according to the molecular size and characteristic structure. For example, 13 PGRPs were found in *Drosophila melanogaster*, of which seven (SA, SB1, SB2, SC1a, SC1b, SC2, and SD) were type PGRP-S and contained signal peptides but no transmembrane domains. The other six were PGRP-L, which are divided into two categories: those in one category contain signal peptides and transmembrane domains, whereas members of the other contain no signal peptides nor transmembrane domains [24]. Sequence analysis revealed that *OfPGRP-A* and *OfPGRP-B* have conserved PGRP domains. *OfPGRP-A* has a signal peptide but no transmembrane domain, whereas *OfPGRP-B* has neither a transmembrane domain nor a signal peptide. We predicted that both genes belong to the short form of PGRP, which is a secreted extracellular protein. For most PGRPs that have amidase activity, this activity is contained in the H–Y–H–T–C structure [25]. Sequence comparison showed that in the *OfPGRP-A* sequence, tyrosine is mutated to serine and cysteine is mutated to serine, suggesting that *OfPGRP-A* lacks amidase activity. *OfPGRP-B* retains five key amidase active sites of H–Y–H–T–C. It also has arginine residues in the DAP-type PGN recognition site, indicating that this gene has amidase activity. *OfPGRP-B* may be involved in recognizing Gram-negative bacteria, leading to activation of the Imd signaling pathway. *OfPGRP-A* and *OfPGRP-B* clustered with the PGRP genes of *P. xylostella* and *M. sexta*, respectively, indicating that PGRP has relatively conserved evolutionary characteristics in lepidopterans.

PGRPs show significantly different expression levels at various developmental stages as well as tissue-specific expression. The expression levels of *BdPGRP-LCa* and *BdPGRP-LCb* of *Bactrocera dorsalis* increase from the mature larva to early pupal stage and from the late pupal to early adult stage. Both genes are highly expressed in the larval midgut and fat bodies but lower in the gonads. *BdPGRP-SA* exhibits the highest expression in adults, lowest expression in the egg and early larval stages, and high expression in the fat body and midgut [26,27]. *PxPGRP-S1* shows the highest expression in the 4th instar larval stage and pupal stage of *Plutellaxylostella*; lowest expression in the egg, 2nd instar, and adult stages; and highest expression in the fat body among tissues [28]. In this study, *OfPGRP-A* and *OfPGRP-B* showed the highest expression in 4th instar larvae. Before the 4th instar, the expression level gradually increased with age. As the larvae grew, immune function gradually enhanced, and the ability to resist pathogens was strongest in the 4th instar larvae. The midgut is an essential immune response center in the intestine, whereas the fat body synthesizes, stores, detoxifies, and metabolizes toxic substances. Both the midgut and fat body are important immune organs that defend against pathogens [29]. In this study, the expression levels of *OfPGRP-A* and *OfPGRP-B* were significantly higher in the midgut than in other tissues. After inoculation by different bacteria, *OfPGRP-A* and *OfPGRP-B* expression changed significantly in each tissue to different extents. Thus, *OfPGRP* expression may differ between tissues.

Insect PGRPs can recognize PGN in the bacterial cell wall and activate the Toll and Imd pathways [30]. PGRP activates the Toll signaling pathway upon recognition of PGN from Gram-positive bacteria but activates the Imd signaling pathway upon recognition of PGN from Gram-negative bacteria. In *Drosophila*, PGRP-SA can bind to lysine-type PGN and activate the Toll pathway [31], whereas PGRP-LE and PGRP-LC cooperate to activate the Imd pathway [32]. In this study, *OfPGRP-A* expression increased significantly after injection of *S. aureus* and *B. thuringiensis*, and *OfPGRP-B* expression increased significantly after injection of *E. coli*. *S. aureus* and *B. thuringiensis* are Gram-positive bacteria with lysine-type PGN, whereas *E. coli* are Gram-negative bacteria with DAP-type PGN. The change in PGRP gene expression after injection with different bacteria indicates that PGRP regulates different signaling pathways. Combined with previous analysis of the gene characteristics, *OfPGRP-A* may be involved in activation of the Toll pathway, and *OfPGRP-B* may be involved in activation of the Imd pathway. In further studies, we will obtain the recombinant proteins of OfPGRP-A and OfPGRP-B using a prokaryotic expression system and perform in vitro experiments to explore the functions of OfPGRP-A and OfPGRP-B proteins, thus determining the possible immune signal pathways in which they participate. This study lays a foundation for further research on the function of PGRP in *O. furnacalis* and will help facilitate the use of entomopathogens for control of this insect pest.

## Figures and Tables

**Table 1:** Primers used in the experiment.

Gene	Forward Primer	Reverse Primer	Purpose
*OfPGRP-A*1	TCAGTACCTGCCGAGGCCAGTC	GAAGGAAGAACCAATGTCCCACCAA	Cloning
*OfPGRP-B*1	TTCATTTCAACAGCGTCAGCCTCG	CGGGTGCGGTGAGTAGTGTTTCC	
*OfPGRP-A*2	ATGTTCCGAAAGTTGAATATTT	GATCGAGCTGACGTCGTCCATC	Full-length clone
*OfPGRP-B*2	ATGCCGGGTCCGCTGCCAGTA	TCATCATAAGTTGCATTCCCCCT	
*OfPGRP-A*3	TGCTGGCCAAAGTCTAGACA	AGTAAGGAACATCGCCCCAA	Real-time PCR
*OfPGRP-B*3	TGGCCGATGAGAGTGTAGTC	GGATACAGTTTTGCGGTGGG	
*RPL18*	ACGGAGGTGGTAACCATCAACA	ACGCCTCCTTCTTGGTGTCG	

## Data Availability

The data presented in this study are available on reasonable request from the corresponding author.
